# *Plasmodium falciparum* induces Foxp3hi CD4 T cells independent of surface *Pf*EMP1 expression via small soluble parasite components

**DOI:** 10.3389/fmicb.2014.00200

**Published:** 2014-05-01

**Authors:** Anja Scholzen, Brian M. Cooke, Magdalena Plebanski

**Affiliations:** ^1^Department of Immunology, Monash UniversityMelbourne, VIC, Australia; ^2^Department of Medical Microbiology, Radboud University Medical CentreNijmegen, Netherlands; ^3^Department of Microbiology, Monash UniversityClayton, VIC, Australia

**Keywords:** Malaria, *Plasmodium falciparum*, regulatory T cell, Foxp3, *Pf*EMP-1, hemozoin

## Abstract

Elevated levels of regulatory T cells following *Plasmodium* infection are a well-reported phenomenon that can influence both protective and pathological anti-parasite responses, and might additionally impact on vaccine responses in acutely malaria infected individuals. The mechanisms underlying their induction or expansion by the parasite, however, are incompletely understood. In a previous study, *Plasmodium falciparum* infected red blood cells (iRBCs) were shown to induce effector-cytokine producing Foxp3int CD4+ T cells, as well as regulatory Foxp3hi CD4+ T cells *in vitro*. The aim of the present study was to determine the contribution of parasite components to the induction of Foxp3 expression in human CD4+ T cells. Using the surface *Pf*EMP1-deficient parasite line 1G8, we demonstrate that induction of Foxp3hi and Foxp3int CD4+ T cells is independent of *Pf*EMP1 expression on iRBCs. We further demonstrate that integrity of iRBCs is no requirement for the induction of Foxp3 expression. Finally, transwell experiments showed that induction of Foxp3 expression, and specifically the generation of Foxp3hi as opposed to Foxp3int CD4 T cells, can be mediated by soluble parasite components smaller than 20 nm and thus likely distinct from the malaria pigment hemozoin. These results suggest that the induction of Foxp3hi T cells by *P. falciparum* is largely independent of two key immune modulatory parasite components, and warrant future studies into the nature of the Foxp3hi inducing parasite components to potentially allow their exclusion from vaccine formulations.

## INTRODUCTION

Malaria caused by infection with protozoan *Plasmodium* parasites is a life threatening disease that is at least partially immune mediated. Disease-contributing factors include excessive inflammatory responses and overwhelming parasite replication insufficiently controlled by anti-parasite immune responses. Regulatory T cells (Tregs) can suppress both protective as well as pathological adaptive immune responses, and are elevated in both human *falciparum* and *vivax* malaria as well as in murine malaria models (Scholzen et al., 2010). Although the consequences of elevated Treg levels during malaria are yet to be determined, several studies indicate that depending on the stage of infection, increased Treg levels can be protective or detrimental to the host (Finney et al., 2010; Hansen and Schofield, 2010; Scholzen et al., 2010). In addition to potentially limiting responses to parasite antigens (Ho et al., 1986; Bejon et al., 2007), elevated Treg levels during acute blood-stage malaria infection might also contribute to the reduced acquisition of immune responses to heterologous antigens, such as standard childhood vaccines (Greenwood et al., 1972; Williamson and Greenwood, 1978; Whittle et al., 1984). The mechanisms underlying the elevated levels of this important cell type during malaria, however, are incompletely understood.

We have previously dissected the host immune mechanisms contributing to the induction of effector-cytokine producing CD25+Foxp3int CD4+ T cells, as well as regulatory CD25+Foxp3hi CD4+ T cells that inhibited Foxp3int effector cytokine production, following *P. falciparum*-infected red blood cells (iRBCs) exposure *in vitro* (Scholzen et al., 2009). The parasite factors responsible for Treg induction during malaria are yet unknown. This is especially relevant as the identification of parasite-specific components relevant for Treg induction may allow the development of intervention strategies directly targeted at the parasite, and the specific exclusion or inclusion of parasite components in therapeutic or vaccine formulations (Casares and Richie, 2009; Higgins et al., 2011).

Two key parasite components that have attracted attention as modulators of immune response in both human and murine malaria are the virulence factor and variant surface antigen *P. falciparum* erythrocyte membrane protein (*Pf*EMP)-1, and the heme degradation product and malaria pigment hemozoin. Whilst findings for *Pf*EMP-1 are controversial, both have been shown in a number of studies to interfere with the activation and maturation of antigen-presenting cells such as monocytes and dendritic cells (Millington et al., 2006; Wykes and Good, 2008; Stevenson et al., 2011). This is particularly relevant as both in human *in vitro* studies (Scholzen et al., 2009; Finney et al., 2012; Clemente et al., 2013) and in murine models (Hisaeda et al., 2008), antigen-presenting cells are crucial mediators of Treg induction and activation by malaria parasites. The aim of the present study was therefore to determine the contribution of parasite components to the induction of Foxp3 expression in human CD4+ T cells.

## MATERIALS AND METHODS

### *P. falciparum* CULTURE AND TROPHOZOITE ISOLATION

Mycoplasma-free blood-stage parasites of *P. falciparum* (strain 3D7) were maintained in O+ erythrocytes in RPMI-1640 medium (JRH, Lenexa, KS, USA) supplemented with 1 mM glutamine, 11 mM glucose, 25 mM HEPES, 0.2% (w/v) sodium bicarbonate, 200 mM hypoxanthine, 40 mg/ml gentamycin (all Sigma–Aldrich, St. Louis, MO, USA), and 0.5% (w/v) AlbuMAX II (GIBCO, Invitrogen, Carlsbad, CA, USA) at 37°C in an atmosphere of 5% CO2 and 1% O2 in N2. Knob-expressing parasites were enriched weekly using gelofusine solution (Braun Melsungen, Germany). The 3D7-derived SBP-1 knock-out parasite line [clone 1G8 (Cooke et al., 2006)] was grown under drug-pressure (2.5 nM WR99210 and 4 μM Ganciclovir). Trophozoite stage parasites were isolated by density gradient centrifugation following layering onto a gradient of 40/60/80% isotonic Percoll (Amersham Biosciences, Uppsala, Sweden). The percentage of infected erythrocytes was typically 90–100%.

### PBMC ISOLATION AND IRBC:PBMC CO-CULTURE

To examine the induction of Foxp3 expression by iRBCs, we employed the *in vitro* co-culture system previously validated in our laboratory (Scholzen et al., 2009). Peripheral blood mononuclear cells (PBMCs) were recovered by Ficoll–Hypaque (Amersham Biosciences) density gradient centrifugation from buffy coats [Australian Red Cross Blood Service (ARCBS), Melbourne, VIC, Australia]. The ARCBS received informed consent from all donors to use their donation for research purposes and the Monash University Human Research Ethics Committee approved the research purpose for which buffy coats were used. Autologous human serum (HS) was obtained by coagulating platelet-rich plasma from buffy coats with 0.3% (w/v) CaCl2, followed by heat inactivation at 56uC for 30 min. PBMCs were cultured in AIM-V medium (GIBCO, Invitrogen) supplemented with 5% autologous HS alone (untreated controls), with non-infected control erythrocytes or trophozoite-stage iRBCs. An iRBC:PBMC ratio of 2:1 was chosen (Scholzen et al., 2009), calculated to reflect a clinically relevant parasitemia found in natural infections (0.1% parasitemia or 5000 iRBC/μl blood) (Minigo et al., 2009; Walther et al., 2009). To obtain iRBC lysate, the integrity of iRBCs was disrupted by five rounds of freeze-thawing. In some experiments, tissue culture inserts (Anopore, NUNC, Naperville, IL, USA) were used to separate iRBCs (inside) from PBMCs (outside the transwell). A pore size of 20 nm was chosen to prohibit transfer of hemozoin crystals, which are on all sides larger than this cut-off (Noland et al., 2003).

### CELL PHENOTYPING BY FLOW CYTOMETRY

**FIGURE 1:**
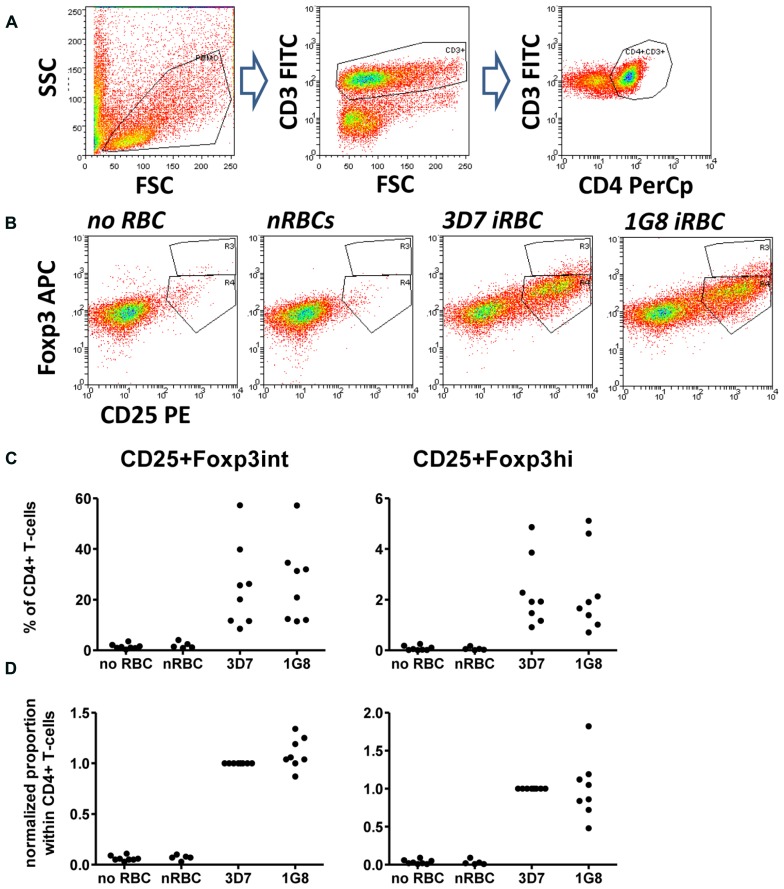
**Infected red blood cell (iRBC)-mediated Foxp3 expression is independent of surface *Pf*EMP-1 expression.** PBMC were cultured alone or in the presence of normal (nRBC) or infected RBCs (iRBC) at an RBC:PBMC ratio of 2:1. RBCs were infected with either the parental strain 3D7 or the SBP-1 knock-out parasite line 1G8. On day 6, cells were harvested and analyzed by flow cytometry. **(A)** Lymphocytes were selected by FSC/SSC gating and further gated based on CD3 and CD4 staining. CD4+ T cells were then analyzed based on CD25 and Foxp3 expression. **(B)** Representative dots plots for PBMC from one donor stimulated with 3D7 and 1G8 parasites. **(C)** Proportions of Foxp3hi and Foxp3int CD4+CD25+ T cells determined as a percentage of CD3+CD4+ T cells for eight donors in six independent experiments. **(D)** Values of all conditions were normalized for each individual donor on proportions induced by 3D7 iRBCs. Vertical bars represent median values.

Cells were washed with PBS and incubated with antibodies diluted in PBS/10% HS/0.01% NaN3 (sodium azide) for 30 min on ice. Surface antibodies were anti-CD4 PerCp (clone SK3), CD3 FITC (clone UCHT1), and CD25 PE (clone M-A251, all BD Biosciences). Intracellular staining with anti-Foxp3 APC (clone PCH101, eBiosciences) was performed using the eBioscience fixation/permeabilization buffer kit. A minimum of 10^5^ events in the lymphocyte gate was acquired using a FACScalibur flow cytometer for 4-color analysis and analyzed using WEASEL software (WEHI, Melbourne, VIC, Australia). Cells were gated first based on forward and side scatter to excluded dead cells and cell debris. T cells in the lymphocyte gate were identified based on CD3 expression, further sub-gated on CD4+ T cells (**Figure 1A**) and CD25+ cells then subdivided into Foxp3hi and Foxp3int cells (**Figure 1B**).

### DATA PRESENTATION AND STATISTICAL ANALYSIS

We employed normalization onto control conditions for each donor, to be able to analyze changes in Foxp3hi or Foxp3int proportions (measured as percentage of CD4 T cells). Normalized values are referred to as fold change compared to control conditions (value 1).

Statistical analysis was carried out using GraphPad Prism software v4 (San Diego, CA, USA). Due to the small power of non-parametric tests to detect differences in small sample sizes, all tests were chosen to be parametric. *P* values between two groups were determined by two-tailed paired Student’s *t*-test. Three or more groups were compared by repeated-measures one-way ANOVA, followed by Tukey’s multiple comparison post test. A *p* < 0.05 was considered significant.

## RESULTS

We firstly addressed the question, whether the induction of Foxp3 expression in CD4+ T cells was dependent on interactions between iRBC surface-expressed *Pf*EMP-1 and corresponding receptors on peripheral blood mononuclear cells such as monocytes using a recently established 3D7-derived surface *Pf*EMP1 deficient parasite line. This parasite line lacks, due to targeted gene disruption, expression of skeleton-binding protein 1 (SBP-1), a Maurer’s cleft-associated protein essential for the transport of *Pf*EMP-1 to the iRBC surface (Cooke et al., 2006). When co-cultured with PBMC for 6 days, surface *Pf*EMP-1 deficient 1G8 parasites induced proportions of CD25+Foxp3hi and CD25+Foxp3int cells that were not significantly different from those induced by wild type 3D7 iRBCs (**Figure 1**, one-way ANOVA with Tukey’s post-test). This indicates that induction of Foxp3 expression in CD4+ T cells is independent of surface *Pf*EMP-1 expression on iRBCs and therefore surface *Pf*EMP-1-host immune receptor interaction.

**FIGURE 2:**
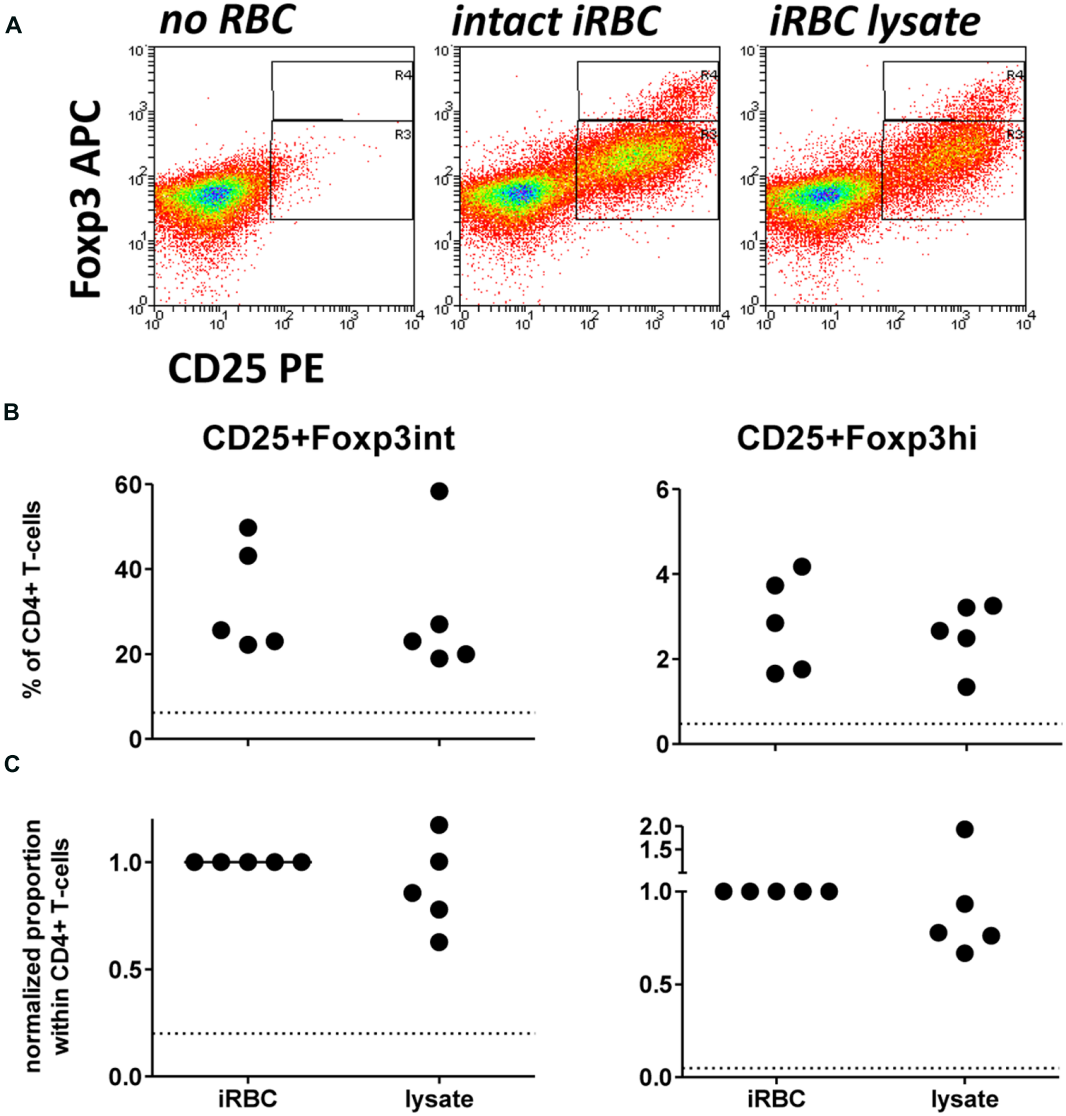
**iRBC-mediated Foxp3 expression does not require intact iRBCs.** PBMC were cultured alone or in the presence of normal (nRBC) or infected RBCs (iRBC) at an RBC:PBMC ratio of 2:1. PBMC were co-cultured with equal amounts of either intact 3D7 iRBCs, or 3D7 iRBC lysate resulting from five rounds of freezing and thawing. **(A)** Representative dots plots for PBMCs from one donor stimulated with intact iRBCs versus iRBC lysate. **(B)** Proportions of Foxp3hi and Foxp3int CD4+CD25+ T cells were determined on day 6 as a percentage of CD3+CD4+ T cells for five donors in two independent experiments. **(C)** Values of all conditions were normalized for each individual donor on proportions induced by intact iRBCs. Vertical bars represent median values. Dotted lines show the upper 95% confidence interval of the mean of uRBC-stimulated control cultures.

Since two previous studies have used iRBC lysate or soluble extract for Foxp3 induction *in vitro* (Finney et al., 2012; Clemente et al., 2013), we first assessed there was a difference between intact and lysed iRBC in their ability to induce CD25+Foxp3 expressing CD4+ T cells. When comparing Foxp3 induction in PBMC co-cultures with either intact iRBCs or iRBC lysate, iRBC lysate was nearly as effective in inducing both CD25+Foxp3hi and CD25+Foxp3int CD4+ T cells (**Figure 2**), with no significant difference between the two for neither absolute percentages or after normalization. Therefore, iRBC integrity is indeed not a prerequisite for the Foxp3 induction in CD4+ T cells upon parasite exposure.

**FIGURE 3:**
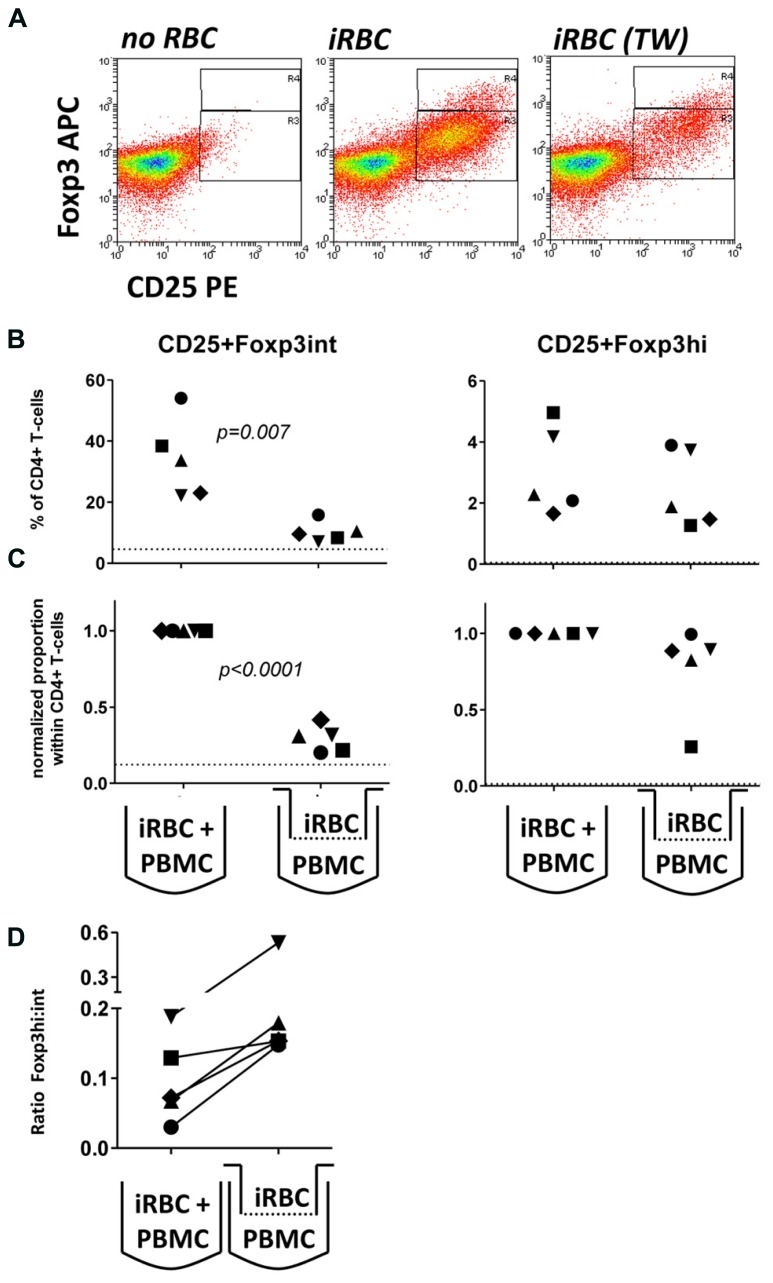
**Foxp3hi and to a lesser degree Foxp3int induction is mediated by soluble iRBC components smaller than 20 nm.** To exclude large membrane fragments and intact hemozoin crystals and to examine the contribution of small soluble molecules derived from iRBCs to Foxp3 induction, intact iRBC were separated from PBMC through a transwell membrane (pore size 20 nm). **(A)** Representative dots plots for PBMCs from one donor cultured in direct contact with iRBCs compared to transwell-separated iRBCs. **(B)** Proportions of Foxp3hi and Foxp3int CD4+CD25+ T cells were determined on day 6 as a percentage of CD4+ T cells for five donors in two independent experiments. **(C)** Values of all conditions were normalized for proportions induced by direct co-culture with iRBCs for each donor. Vertical bars represent median values. **(D)** The ratio of Foxp3hi:Foxp3int cells within the CD4+CD25+ T cell population was calculated for each individual donor in direct or transwell separated co-cultures. Vertical bars represent median values. Individual donors are identified by unique symbols. Dotted lines show the upper 95% confidence interval of the mean of uRBC-stimulated control cultures.

To further investigate the possibility that large intracellular components such as hemozoin crystals interacting with monocytes were contributing to Foxp3 induction, we employed tissue culture inserts to separate iRBCs (inside) and PBMCs (outside the transwell). We specifically chose a pore size of 20 nm to prohibit transfer of large parasite components, including membrane fragments and intact hemozoin crystals, which are larger than 20 nm in diameter on either side of their brick-like cuboidal body (Noland et al., 2003), between the two chambers. Accordingly, in this transwell setting we found no light-microscopic evidence of hemozoin incorporation into monocytes (which in direct co-cultures are typically filled with dark hemozoin material, data not shown). As shown in **Figure 3**, similar to direct co-cultures, iRBC separated from PBMC by a transwell were also capable of inducing Foxp3 expression in CD4+ T cells. Importantly, in four out of five donors Foxp3hi CD4+ T cells were still induced at levels comparable to direct co-culture (**Figure 3**), while induction of Foxp3int CD4+ T cells was reduced in all five donors tested (**Figures 3B,C**); 20–31% compared to direct co-culture; *p* < 0.001, one-way ANOVA with Tukey’s post-test). As a result, there was a trend that exposure of PBMC to soluble iRBC components smaller than 20 nm instead of complete iRBC enhanced the ratio of Foxp3hi:Foxp3int cells within the CD4+CD25+ population (**Figure 3D**).

## DISCUSSION

In this study, we show that *P. falciparum* iRBCs can induce Foxp3hi CD4 T cells independent of surface-expressed PfEMP-1 via soluble parasite components smaller than 20 nm.

Similar to our finding that induction of Foxp3 expression is independent of iRBC surface *Pf*EMP-1 expression and contact with intact iRBCs, a recent study demonstrated that human monocyte-derived DC maturation can be inhibited by *P. falciparum* independent of surface *Pf*EMP1 expression and also across a transwell (Elliott et al., 2007). Using a murine malaria model, Orengo et al. (2008) reported that inhibition of murine DC maturation following *P. yoelii* infection was also mediated by a yet unidentified soluble factor. Future studies are now needed to further identify the soluble factor mediating these immunomodulatory effects of the parasite. Importantly, monocytes or monocytes-derived DCs were previously shown to be required to drive the induction of Foxp3 expression in human CD4 T cells upon *P. falciparum* exposure *in vitro* (Scholzen et al., 2009; Clemente et al., 2013) and DCs activated Tregs in *P. yoelii* infected mice (Hisaeda et al., 2008). Future studies may therefore address the question whether it may even be the same mechanism by which the parasite modulates not only with monocyte and DC function, but also initiates the induction of Tregs.

Previous studies have used either intact iRBCs (Scholzen et al., 2009), iRBC lysate (Finney et al., 2012), or the soluble fraction of iRBC lysate (Clemente et al., 2011) as a stimulus for Foxp3 expression in human CD4 T cells *in vitro*. In this direct side-by-side comparison we show that Foxp3 induction by iRBC lysate is indeed comparable to intact iRBC. Therefore, if direct cell–cell interaction between immune cells and iRBCs are not a pre-requisite for Treg induction, which is instead mediated by soluble factors resulting from schizont rupture, then Treg induction can also occur at distant sites clear of measurable parasitemia.

For instance, while mature trophozoite-stage *P. falciparum* parasites are typically sequestered in the microvasculature (to avoid clearance in the spleen), soluble parasite components released upon parasite rupture would have access to monocytes and T cells in the spleen. Moreover, soluble parasite components might also mediate Treg induction in more distant sites outside the circulation, such as in lymph nodes. In as how far this occurs, and whether this would affect vaccination-induced immune responses during acute blood-stage malaria infection (Greenwood et al., 1972; Williamson and Greenwood, 1978; Whittle et al., 1984), remains to be determined. Finally, such small parasite components may be able to cross the placental barrier and thus explain and contribute to the induction of Tregs in cord blood even in the absence of direct cord blood parasitemia. Indeed, in some studies levels of Tregs have been found to be elevated in neonates born to mothers who had experienced malaria episodes during pregnancy (Brustoski et al., 2006; Mackroth et al., 2011), while in other studies, *ex vivo* cord blood Treg frequencies were unaffected by placental malaria and only increased only upon *in vitro* stimulation with iRBC extract (Flanagan et al., 2010; Soulard et al., 2011). It is yet unclear whether Treg induction during a single *Plasmodium* infection predisposes the immune system to heightened regulatory responses at the next encounter with the parasite, but *in utero* exposure to Treg-inducing parasite components may prime the fetus’s immune system to respond with a less inflammatory response upon re-exposure to malaria-antigens (Malhotra et al., 2009; Flanagan et al., 2010). Moreover, such malaria-induced immune modulation might also be one explanation for observations of reduced vaccination-responses in children born to women with placental-malaria (Labeaud et al., 2009; Walther et al., 2012).

Hemozoin has been shown in several studies to activate TLR9 (Shio et al., 2010). Moreover, DCs activated via TLR9 [either by iRBCs in mice (Hisaeda et al., 2008) or CpG DNA in human (Moseman et al., 2004)] can induce/activate Tregs. These findings indicate a potential role for hemozoin in Treg induction. It remains to be formally shown whether iRBC-purified or synthetic hemozoin contributes to the induction of Tregs by *P. falciparum* iRBCs. Our results from experiments using transwell inserts with a 20 nm pore size to separate PBMCs and iRBCs, however, suggest that hemozoin crystals do not have a major contribution to the generation of Foxp3hi T cells: Intact hemozoin crystals are larger than 20 nm (Noland et al., 2003) and therefore unlikely to have crossed the 20 nm sized pores of the transwell inserts used in this study. We adoped a transwell approach to prohibit the transfer of intact hemozoin crystals after schizont rupture, since it is currently technically not possible to deplete iRBC extracts of hemozoin without denaturing other lysate components and antigens (Coban et al., 2010). And while we cannot rule out that smaller hemozoin crystal fragments might have crossed the transwell border, the TLR9 binding capacity of hemozoin has recently been shown to be restricted to a crystal size range of 50–200 nm, while hemin molecules smaller than 50 nm were ineffective (Coban et al., 2010).

A curious finding was the trend towards a favored induction of Foxp3hi over Foxp3int T cells when components larger than 20 nm were excluded from the co-culture using transwell membranes. Whilst these data require further analysis in future studies, it is tempting to speculate that small soluble parasite components are preferentially driving Foxp3hi induction, whilst Foxp3int effector T cells may rely on a greater pool of membrane associated antigens. We have previously shown that although iRBC-mediated induction of Foxp3hi T cells does rely on T effector-produced IL-2 and is further driven by cytokines such as IL-10 and TGFβ, in itself it is not dependent on MHC class II antigen-presentation and those cells therefore not necessarily malaria antigen-specific (Scholzen et al., 2009). The current data are in line with this finding, suggesting that activation of T cells in an environment of direct contact of antigen-presenting cells with intact iRBCs might result in a more efficient induction of (Foxp3int) effector T cells in a parasite antigen-specific manner. In contrast, induction of Foxp3hi T cells with a T regulatory phenotype may also occur independent of membrane-associated antigens at a distant site, mediated by cytokines acting in concert with circulating soluble parasite molecules. Further research is now necessary to determine the nature and mechanism of action of these soluble parasite components.

To conclude, our results indicate that the induction of Foxp3hi regulatory T cells by *P. falciparum* may be largely independent of two key immunomodulatory parasite components, namely the surface protein *Pf*EMP1 and the malaria pigment hemozoin and warrant future studies into the nature of the Foxp3hi inducing parasite components. Furthermore, similar to their distinct cytokine requirements (Scholzen et al., 2009), Foxp3int effector-like and Foxp3hi regulatory-like CD4+ T cells appear to rely on different parasite components for their induction. These findings merit future in-depth studies to identify the parasite components responsible for regulatory versus effector T cell induction. Identification of such parasite components will be important to ensure appropriate exclusion or inclusion of such parasite components from vaccine formulations.

## AUTHOR CONTRIBUTIONS

Conceived and designed the experiments: Anja Scholzen, Magdalena Plebanski. Performed the experiments and analyzed the data: Anja Scholzen. Contributed essential reagents/materials/analysis tools: Brian M. Cooke, Magdalena Plebanski. Interpreted data and wrote the paper: Anja Scholzen, Magdalena Plebanski.

## Conflict of Interest Statement

The authors declare that the research was conducted in the absence of any commercial or financial relationships that could be construed as a potential conflict of interest.
